# Genotyping and molecular profiling of intestinal microsporidiosis and cryptosporidiosis in HIV-infected patients in Alborz Province, Iran

**DOI:** 10.1186/s13099-025-00785-2

**Published:** 2025-12-11

**Authors:** Benyamin Djawadi, Nazila Parvizi, Hossein Vazini, Milad Badri, Aida Vafae Eslahi, Ioannis Adamopoulos, Mahendra Pal, Majid Pirestani

**Affiliations:** 1 Department of Parasitology, Faculty of Medical Sciences, Tarbiat Modares University, Tehran, Iran; 2 Department of Nursing, Ha.C, Islamic Azad University, Hamedan, Iran; 3 Medical Microbiology Research Center, Qazvin University of Medical Sciences, Qazvin, Iran; 4 Department of Public Health Policy, Sector of Occupational and Environmental Health, School of Public Health, University of West Attica, Athens, Greece; 5 Narayan Consultancy on Veterinary Public Health and Microbiology, Saphire Lifestyle, Bharuch, 392012 Gujarat India

**Keywords:** Cryptosporidium parvum, Cryptosporidium hominis, Enterocytozoon bieneusi, Encephalitozoon intestinalis, HIV/AIDS, Gastrointestinal symptoms, Genotyping, Zoonotic transmission

## Abstract

**Background:**

*Cryptosporidium* and Microsporidia are major opportunistic pathogens in individuals with HIV, frequently causing gastrointestinal manifestations. Molecular identification of these parasites provides crucial insights into their transmission dynamics, clinical relevance, and zoonotic potential.

**Methods:**

This cross-sectional study investigated 275 HIV-infected patients in Alborz Province, Iran (2018–2020). Stool samples were examined using Ziehl–Neelsen and modified trichrome staining, followed by PCR amplification and sequencing of the 18 S rRNA and GP60 genes for *Cryptosporidium* spp., and the ITS region for *Enterocytozoon bieneusi* and *Encephalitozoon intestinalis*. Associations between parasitic infections and demographic/clinical variables were analyzed using univariate and multivariable methods.

**Results:**

Molecular analysis identified *Cryptosporidium* spp. in 7.6% and Microsporidia in 9.1% of patients, including *E. bieneusi* (6.5%), *E. intestinalis* (2.5%), and mixed infections (1.8%). Subtyping revealed that *C. parvum* (5.8%) predominantly belonged to subtype family IId (IIdA20G1, IIdA19G1), while *C. hominis* (1.8%) was IdA15G1. *E. bieneusi* genotypes D, Peru6, and J were detected—genotype J being reported for the first time in Iranian HIV-positive patients. Infections were significantly associated with clinical symptoms including chronic diarrhea, abdominal pain, vomiting, and fever. The highest rates of infection were found among patients with CD4 + counts < 200 cells/µL, no history of ART, animal contact, and use of well water.

**Conclusions:**

This study highlights the clinical and epidemiological significance of *Cryptosporidium*, *E. bieneusi*, and *E. intestinalis* in HIV-infected individuals. The identification of zoonotic genotypes and their association with gastrointestinal symptoms and immunosuppression emphasizes the need for routine molecular screening, targeted public health interventions, and adoption of One Health strategies to control transmission.

## Introduction

Diarrhea is a major contributor to morbidity in human immunodeficiency virus (HIV)-infected patients both in developing and developed countries, affecting almost 40% of the people who die from the disease [1, 2]. Microsporidiosis and Cryptosporidiosis are opportunistic and the most prevalent parasitic infections causing chronic diarrhea in immunodeficiency conditions, especially HIV/acquired immunodeficiency syndrome (AIDS) [3, 4].

The occurrence of microsporidia and *Cryptosporidium* is significantly higher in HIV + patients with chronic diarrhea and CD4 + lymphocyte counts of less than 200 cells/mm³ [5]. Microsporidia parasites are obligate intracellular pathogens that infect various vertebrates and invertebrates globally [6, 7]. Currently, more than 200 genera and 1500 species of microsporidia have been described, and 17 of those species have been associated with human diseases [8].

Microsporidiosis is caused primarily by two microsporidian species, *Enterocytozoon bieneusi* and *Encephalitozoon intestinalis* [4]. Among these, *E. bieneusi* is the most frequently detected species in humans and was first identified in AIDS patients in 1985 [9]. Since then, *E. bieneusi* has been recognized as an important opportunistic pathogen in individuals with compromised immunity, including HIV/AIDS patients, cancer patients, organ transplant recipients, as well as in children, the elderly, and travelers [8]. It has been reported from a wide range of hosts—humans, livestock, birds, and wildlife and isolated from diverse environmental sources such as wastewater, drinking water, recreational waters, coastal areas, raw milk, and fresh vegetables. This wide distribution highlights its potential for both zoonotic and environmental transmission. Moreover, *E. bieneusi* exhibits extensive genetic diversity, with more than 500 genotypes identified so far across different host species and ecological settings, underscoring its adaptive capacity and epidemiological complexity [9].

Studies on *E. bieneusi* genotypes are commonly based on the analysis of the polymorphism of the internal transcribed spacer (ITS) nucleotide sequences. Presently, more than 1600 full-length *E. bieneusi* ITS sequences from more than 650 studies have been deposited in GenBank. The most prevalent genotypes of *E. bieneusi* are types IV, EbpC, A, and D. Nevertheless, Peru6, Peru8, Peru11, D, EbpC, and type IV, which are the most common genotypes in humans, have been frequently reported from domestic and wild animals, as well as various water sources, indicating the possibility of zoonotic and waterborne transmission [8].

*Cryptosporidium* is an intracellular protozoan that is recognized as a human and animal pathogen, given its frequency in waterborne outbreaks worldwide [10, 11]. Cryptosporidiosis causes diarrhea in both immunocompetent and immunocompromised humans and animals [12–14]. However, it leads to serious diarrhea which may be fatal in immunocompromised individuals [3, 15]. Among at least 48 species and more than 120 recognized genotypes of *Cryptosporidium* reported worldwide [16], 22 species and two genotypes have been diagnosed in humans [17]. The majority of human cases of cryptosporidiosis are caused by five *Cryptosporidium* species: *Cryptosporidium hominis*, *C. parvum*, *C. meleagridis*, *C. felis*, and *C. canis* [18].

HIV/AIDS is a prominent cause of mortality and morbidity in low- and middle-income countries. Between 1990 and 2019, Iran witnessed a 108% increase in the number of HIV/AIDS-related mortalities, rising from 566 to 1,177 deaths. According to the Joint United Nations Program on HIV/AIDS (UNAIDS), the number of individuals living with HIV/AIDS in Iran in 2019 is expected to be 59,000 cases [19]. The recent increases in HIV/AIDS burden in Iran have important implications for opportunistic infections. As more people live with HIV for longer periods, gaps in care, delayed diagnosis, or incomplete antiretroviral therapy (ART) coverage can lead to increased numbers of immunosuppressed individuals who are susceptible to enteric pathogens. Opportunistic protozoa such as *Cryptosporidium* spp. and microsporidia exploit weakened mucosal and systemic immunity, leading to prolonged diarrheal disease, malabsorption and increased morbidity. Therefore, regional increases in HIV prevalence and HIV-related mortality necessitate strengthened surveillance of enteric opportunistic infections to guide clinical management and public-health interventions.

The aim of the current study was the molecular identification and genotyping of *Enterocytozoon*, *Encephalitozoon*, and *Cryptosporidium* isolates from HIV-infected patients referred to a behavioural diseases consultation centre in Alborz Province, Iran, using ribosomal RNA (rRNA) gene analysis.

## Materials and methods

### Study population and sample collection

This cross-sectional study was conducted between 2018 and 2020 at the Department of Medical Parasitology and Entomology, Tarbiat Modares University, Tehran, Iran. A total of 275 HIV-positive patients (165 males and 110 females), both symptomatic and asymptomatic, who were receiving antiretroviral therapy (ART), were consecutively recruited from all eligible individuals referred to the Counselling Centre for Behavioral Diseases in Alborz Province, Iran. The dates correspond to the period during which consecutive patients were referred to and attended the centre, thus capturing the entire available cohort for molecular screening during the study window.

Demographic and clinical data, including age, gender, and CD4 + T-cell counts, were collected using a predesigned structured questionnaire. Informed consent was obtained from all participants prior to sample collection, and the study protocol was approved by the Ethical Committee of Tarbiat Modares University, Tehran, Iran (IR.MODARES.REC.1397.197). The final sample size (*n* = 275) represents the total number of consenting patients attending the center during the specified period. To ensure robust statistical analysis and minimize the risk of model overfitting, the “events-per-variable” (EPV) principle was applied in multivariable logistic regression, including only predictors with approximately ≥ 10 outcome events per variable. This methodological approach is acknowledged as a limitation and was considered in the interpretation of the results.

Stool specimens were preserved in 2.5% potassium dichromate and stored at 4 °C, then safely transported to the laboratory for microscopic examination. All samples were screened for *Microsporidia* spores using the modified trichrome blue staining method under light microscopy.

### DNA extraction

DNA was extracted from stool specimens using a modified cetyltrimethylammonium bromide (CTAB) method as described before [20].

### Genotyping and molecular identification

Sequencing chromatograms were inspected and edited using Sequencer v5.4.6 [21]. The edited sequences were exported in FASTA format and analyzed using the Basic Local Alignment Search Tool (BLAST) on the NCBI GenBank database to confirm species identity and determine sequence similarity with reference isolates. Multiple sequence alignments were performed using Clustal Omega [Sievers & Higgins, 2014] and verified in BioEdit v7.2.5 [22]. for manual correction of ambiguous bases.

Distinct genotypes and subtypes were identified based on sequence similarity thresholds and characteristic polymorphic loci for the gp60, 18 S rRNA, and ITS regions. Representative sequences from each genotype and subtype were submitted to GenBank under the assigned accession numbers.

#### Microsporidia detection and genotyping

Using specific primers (Metabion, Germany), the genotyping of *E. bieneusi* and *E. intestinalis* was conducted through sequence analysis of the ITS region of the rRNA gene and amplified by the Nested-PCR method.

The PCR step for ITS includes 40 cycles, each consisting of 94 °C for 30 s, 52 °C for 30 s, and 72 °C for 30s. An initial denaturation at 94 °C for 5 min and a final extension at 72 °C for 10 min was included. The employed primers specific to *E. bieneusi* and *E. intestinalis* are included in Table 1.

**Table 1:** Specific primers used in Nested-PCR

Parasite	Gene	Primer	Primer sequence	Ref
*E. bieneusi*	ITS	EBITS3	GGTCATAGGGATGAAGAG TTCGAGTTCTTTCGCGCTC (434 bp) GCTCTGAATATCTATGGCT ATCGCCGACGGATCCAAGTG (389 bp)	[23]
		EBITS4		
		EBITS1		
		EBITS2.4		
*E. intestinalis*	ITS	MSP-1	TGAATGKGTCCCTGT TCACTCGCCGCTACT (~ 350–380 bp) GGAATTCACACCGCCCGTCRYTAT CCAAGCTTATGCTTAAGTYMAARGGGT (~ 280–310 bp)	[24]
		MSP-2 A		
		MSP-3		
		MSP-4 A		
*Cryptosporidium*	18 S rRNA	SHP1	5’-ACCTATCAGCTTTAGACGGTAGGGTAT-3’ 5’-TTCTCATAAGGTGCTGAAGGAGTAAGG-3’ (~ 750 bp) 5’-ACAGGGAGGTAGTGACAAGAAATAACA-3’ 5’-AAGGAGTAAGGAACAACCTCCA-3’ (~ 600 bp)	[25]
		SHP2		
		SHP3		
		SSU-R3		
	GP60	F1	5’-ATAGTCTCCGCTGTATTC-3’ 5’-GCAGAGGAACCAGCATC-3’ (~ 860 bp) 5’-TCCGCTGTATTCTCAGCC-3’ 5’-GAGATATATCTTGGTGCG-3’ (~ 470 bp)	[26]
		R1		
		F2		
		R2		

#### *Cryptosporidium* detection, genotyping and subtyping

The genotype and subtype of *Cryptosporidium* in stool specimens were determined using 10 pM of specific primers (Metabion, Germany) targeting the small subunit ribosomal RNA (18 S rRNA) gene and the 60-kDa glycoprotein (GP60) gene. The PCR protocol for 18 S amplification consisted of an initial denaturation at 94 °C for 5 min, followed by 40 cycles of denaturation at 94 °C for 45 s, annealing at 56 °C for 45 s, and extension at 72 °C for 60 s, with a final extension at 72 °C for 10 min. For the GP60 gene, the cycling conditions followed previously published protocols with minor optimization for annealing temperature and cycle number (see Table 1 for primer sequences). PCR products were visualized by electrophoresis on a 1.5% agarose gel prepared in Tris-acetate-EDTA (TAE) buffer and stained with DNA-safe stain. Positive and negative controls were included in each PCR run to ensure amplification accuracy.

### Nucleotide sequence accession numbers

The nucleotide sequences obtained from the current study were deposited in GenBank under accession numbers OQ786807-OQ786826/OR016769-OR016777/OR036995-OR037003.

### Phylogenetic analysis

The obtained nucleotide sequences were edited and aligned using BioEdit v7.2.6 [22], and Clustal Omega [27]. To improve alignment accuracy, multiple sequence alignments were cross-validated using MAFFT v7 with the L-INS-i algorithm [28]. Poorly aligned regions and terminal gaps were inspected manually, and only homologous positions were retained to ensure consistent sequence length across isolates.

The best-fit nucleotide substitution model for each dataset was determined using the Bayesian Information Criterion (BIC) implemented in MEGA X [29]. The TN93 + G model was selected as the optimal model for the *Enterocytozoon bieneusi* ITS region and both *Cryptosporidium* genes (18 S rRNA and GP60).

Phylogenetic relationships were inferred using Bayesian inference implemented in BEAST v2.7.7 [30].Two independent Markov Chain Monte Carlo (MCMC) runs were performed for 50 million generations, sampling every 5,000 states. The first 10% of samples were discarded as burn-in. Parameter convergence and effective sample sizes (ESS >200) were verified using Tracer v1.7.2 [31]. The resulting tree files from both runs were combined with LogCombiner v2.7.7, and maximum clade credibility (MCC) trees were generated in TreeAnnotator v2.7.7 using mean node heights. All final phylogenetic trees were visualized and annotated in FigTree v1.4.4 [32], with branch support values represented by posterior probabilities (≥ 0.8 considered strong). Each sequence tip label included the isolate code, host species, and geographic origin. Reference sequences representing both human and animal isolates from various geographic regions were retrieved from GenBank for comparative analysis and to infer possible zoonotic and transmission linkages.

### Statistical analysis

The distribution of *Cryptosporidium* and *E. bieneusi* genotypes, as well as their associations with demographic and clinical variables, are summarized in Table 2. Logistic regression analyses were performed to assess associations between infection status and potential risk factors, adjusting for age, sex, and ART status.

**Table 2:** Distribution of *Cryptosporidium* and microsporidia species and subtypes in HIV-positive patients by demographic and clinical risk factors

Patient characteristics (Risk factors)	No. Sample (%)	C. hominis (%)	C. parvum (%)	E. bieneusi (%)	E. intestinalis (%)	Mixed Infection (%)	Subtype C. parvum (*n*)	Genotype E. bieneusi (*n*)
**Age group (years)**								
30≥	73 (26.5)	1 (1.4)	6 (8.2)	4 (5.5)	1 (1.4)	1 (1.4)	A20G1 (4) A15G2R1 (1) A19G1 (1)	D (3) Peru6 (1)
31–40	102 (37.1)	2 (1.9)	5 (4.9)	9 (8.8)	2 (1.9)	3 (2.9)	A20G1 (3) A19G1 (1) A15G2R1 (1)	D (5) Peru6 (3) J (1)
41–50	70 (25.5)	2 (2.9)	3 (4.3)	3 (4.3)	3 (4.3)	1 (1.4)	A20G1 (1) A19G1 (1) A15G2R1 (1)	D (1) Peru6 (1) J (1)
51≤	30 (10.9)	-	2 (6.7)	2 (6.7)	1 (3.4)	-	A20G3R1(1) A19G1(1)	Peru6 (1) J (1)
Total	275 (100)	5 (1.8)	16 (5.8)	18 (6.5)	7 (2.5)	5 (1.8)	A20G1 (8) A19G1 (4) A15G2R1 (3) A20G3R1 (1)	D (10) Peru6 (5) J (3)
**Gender**								
Male	165	4	11	12	5	3	A20G1 (5) A19G1 (4) A15G2R1 (1) A20G3R1 (1)	D (6) Peru6 (3) J(3)
Female	110	1	5	6	2	2	A20G1 (3) A15G2R1 (2)	D (4) Peru6 (2)
**Diarrhea**								
Acute	19	-	2	5	1	3	A20G3R1 (1) A20G1 (1)	D (4) Peru6 (1)
Chronic	28	3	11	9	3	2	A20G1 (7) A19G1 (4)	D (6) J (3)
No	228	2	3	4	3	-	A15G2R1 (3)	Peru6 (4)
**Vomiting**								
No	259	5	13	11	5	1	A20G1 (5) A19G1 (4) A15G2R1 (3) A20G3R1 (1)	D (7) Peru6 (2) J (2)
Yes	16	-	3	7	2	4	A20G1(3)	D (3) Peru6 (3) J (1)
**Abdominal pain**								
No	202	1	3	1	2	-	A20G1 (1) A19G1 (1) A20G3R1 (1)	Peru6 (1)
Yes	73	4	13	17	5	5	A20G1 (7) A19G1 (3) A15G2R1 (3)	D (10) Peru6 (4) J (3)
**Fever**								
No	252	4	11	8	3	1	A20G1 (5) A19G1 (3) A15G2R1 (2) A20G3R1 (1)	D (5) Peru6 (2) J (1)
Yes	23	1	5	10	4	4	A20G1 (3) A19G1 (1) A15G2R1 (1)	D (5) Peru6 (3) J (2)
**Contact with animals**								
No	154	3	2	5	4	-	A20G1 (1) A19G1 (1)	D (1) Peru6 (4)
Yes	121	2	14	13	3	5	A20G1 (7) A19G1 (3) A15G2R1 (3) A20G3R1 (1)	D (9) Peru6 (1) J (3)
**HAART therapy**								
Yes	230	2	6	3	2	-	A20G1 (3) A19G1 (2) A15G2R1 (1)	D (2) Peru6 (1)
No	45	3	10	15	5	5	A20G1 (5) A19G1 (2) A15G2R1 (2) A20G3R1 (1)	D (8) Peru6 (4) J (3)
**Water source**								
Tap water	249	1	8	8	6	2	A20G1 (4) A19G1 (2) A15G2R1 (1) A20G3R1 (1)	D (4) Peru6 (2) J (2)
Well water	25	4	7	9	1	2	A20G1 (3) A19G1 (2) A15G2R1 (2)	D (5) Peru6 (3) J (1)
Surface water	1	-	1	1	-	1	A20G1 (1)	D (1)
**CD4 + cell count**								
CD4 ≥ 200	244	2	5	10	3	1	A20G1 (2) A19G1 (2) A15G2R1 (1)	D (7) Peru6 (3)
CD4 < 200	31	3	11	8	4	4	A20G1 (6) A19G1 (2) A15G2R1 (2) A20G3R1 (1)	D (3) Peru6 (2) J (3)

## Results

A total of 275 HIV-positive patients were examined for *Cryptosporidium* and *Microsporidia* infections. Molecular analysis identified *Cryptosporidium spp.* in 7.6% (21/275) and *Microsporidia spp.* in 9.1% (25/275) of participants. Among them, *C. parvum* was detected in 5.8% (16/275), *C. hominis* in 1.8% (5/275), *E. bieneusi* in 6.5% (18/275), and *E. intestinalis* in 2.5% (7/275). Mixed infections involving more than one parasite were observed in 1.8% (5/275) of patients.

### Clinical correlations

Infections with all three parasites were significantly associated with gastrointestinal symptoms. Among patients with chronic diarrhea (*n* = 28), *C. parvum* was found in 39.3% (11/28), *E. bieneusi* in 32.1% (9/28), and *E. intestinalis* in 10.7% (3/28). Vomiting was reported in 16 patients, 7 of whom tested positive for *E. bieneusi*, 3 for *C. parvum*, and 2 for *E. intestinalis*. Abdominal pain and fever were also more frequent among infected individuals. Of those with abdominal pain (*n* = 73), 23.3% were infected with *C. parvum*, 17.8% with *E. bieneusi*, and 6.8% with *E. intestinalis*.

### Associations with immunological and environmental factors

A strong inverse correlation was observed between CD4 + T-cell count and infection prevalence. Among patients with CD4 + counts < 200 cells/µL (*n* = 31), *C. parvum* was detected in 35.5% (11/31), *E. bieneusi* in 25.8% (8/31), and *E. intestinalis* in 12.9% (4/31), significantly higher than those with CD4 + ≥ 200 (*p* < 0.001). Additionally, patients not receiving highly active antiretroviral therapy (HAART) (*n* = 45) exhibited markedly higher infection rates compared to those on therapy: *C. parvum* (22.2% vs. 2.6%), *E. bieneusi* (33.3% vs. 1.3%), and *E. intestinalis* (11.1% vs. 0.9%).

Environmental exposure also played a significant role. Among patients with contact with animals (*n* = 121), prevalence of *C. parvum*, *E. bieneusi*, and *E. intestinalis* was 11.6%, 10.7%, and 2.5%, respectively. Use of well water (*n* = 25) was associated with higher detection rates for *C. hominis* (16%), *E. bieneusi* (36%), and *E. intestinalis* (4%), suggesting waterborne transmission.

### Multivariate logistic regression

To determine the independent predictors of infection, a multivariable logistic regression analysis was performed. The results showed that a CD4 + T-cell count below 200 cells/µL was a strong independent risk factor for infection with both *C. parvum* (adjusted odds ratio [aOR] = 8.4; 95% confidence interval [CI]: 3.2–21.7; *p* < 0.001) and *E. bieneusi* (aOR = 6.5; 95% CI: 2.1–19.6; *p* = 0.002). Additionally, lack of antiretroviral therapy (ART) significantly increased the likelihood of infection with *E. bieneusi* (aOR = 5.9; 95% CI: 1.8–19.1; *p* = 0.004) and *E. intestinalis* (aOR = 4.3; 95% CI: 1.1–17.3; *p* = 0.038). Contact with animals was independently associated with a higher risk of *C. parvum* infection (aOR = 3.7; 95% CI: 1.4–9.5; *p* = 0.007), further suggesting a zoonotic route of transmission. Moreover, use of well water emerged as a significant predictor for *C. hominis* infection (aOR = 6.8; 95% CI: 1.9–24.5; *p* = 0.003), indicating a possible role of contaminated water sources. Other demographic factors, including age, sex, and education level, were not found to be independently associated with infection in the final regression model.

### Molecular detection, genotyping, subtyping and phylogenetic analysis

To confirm species and genotype identification and explore the genetic relationships among isolates, Bayesian phylogenetic analyses were conducted using BEAST v2.7.7 under the TN93 + G substitution model. Analyses were performed separately for the *Cryptosporidium* 18 S rRNA and gp60 genes, and the *Enterocytozoon bieneusi* ITS region. Two independent MCMC runs of 50 million generations were conducted for each dataset, sampling every 5,000 generations, and the first 10% of trees were discarded as burn-in. Posterior probabilities ≥ 0.8 were considered statistically well supported. The resulting Maximum Clade Credibility (MCC) trees were visualized and annotated in FigTree v1.4.4, including isolate codes, host origins, and geographical locations.

The *C. parvum* isolates belonged mainly to subtype family IId, including IIdA20G1 (*n* = 8), IIdA19G1 (*n* = 4), and one IIdA20G3R1 isolate. Three IIaA15G2R1 subtypes were also detected. All five *C. hominis* isolates were classified as IdA15G1. For *E. bieneusi*, three genotypes were identified: D (*n* = 10), Peru6 (*n* = 5), and J (*n* = 3). Genotype J was reported for the first time in HIV-positive individuals in Iran.

**Fig. 1:**
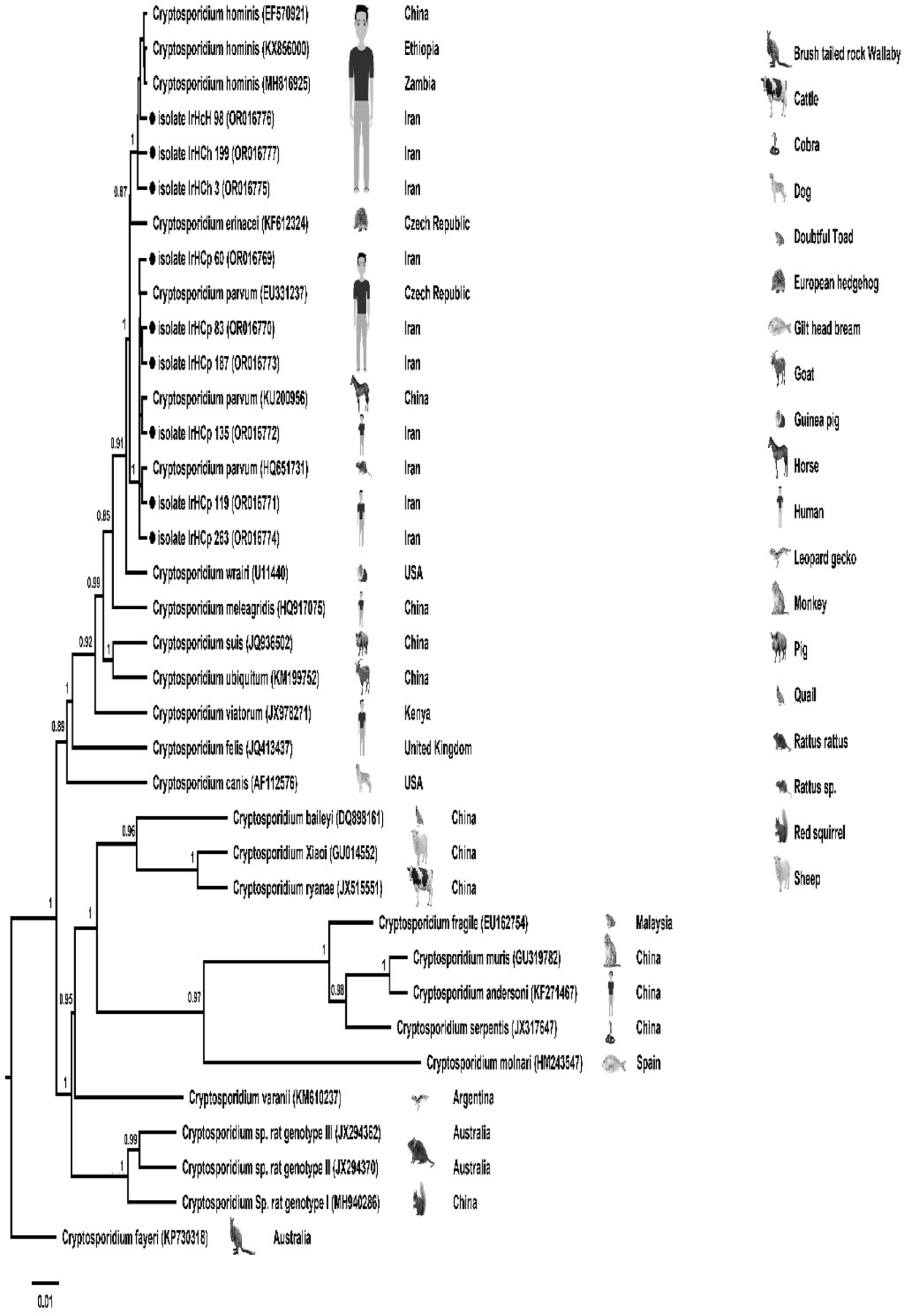
Bayesian phylogenetic tree based on partial 18 S rRNA gene sequences of *Cryptosporidium* spp. constructed in BEAST v2.7.7 under the TN93 + G model. Posterior probabilities (≥ 0.8) are shown at the nodes. The tree reveals clear separation of *C. parvum* and *C. hominis* clusters. Iranian isolates (●) grouped within their respective species, showing close genetic similarity to reference sequences from GenBank. Scale bar represents the number of substitutions per site

The Bayesian phylogenetic tree of *Cryptosporidium* spp. constructed using 18 S rRNA gene sequences showed clear separation of species into distinct clades. Iranian isolates clustered mainly with *C. parvum* and *C. hominis*, consistent with the molecular typing results and clinical findings. High posterior probability values supported species-level identification, validating the accuracy of molecular diagnostics (Fig. 1).

**Fig. 2:**
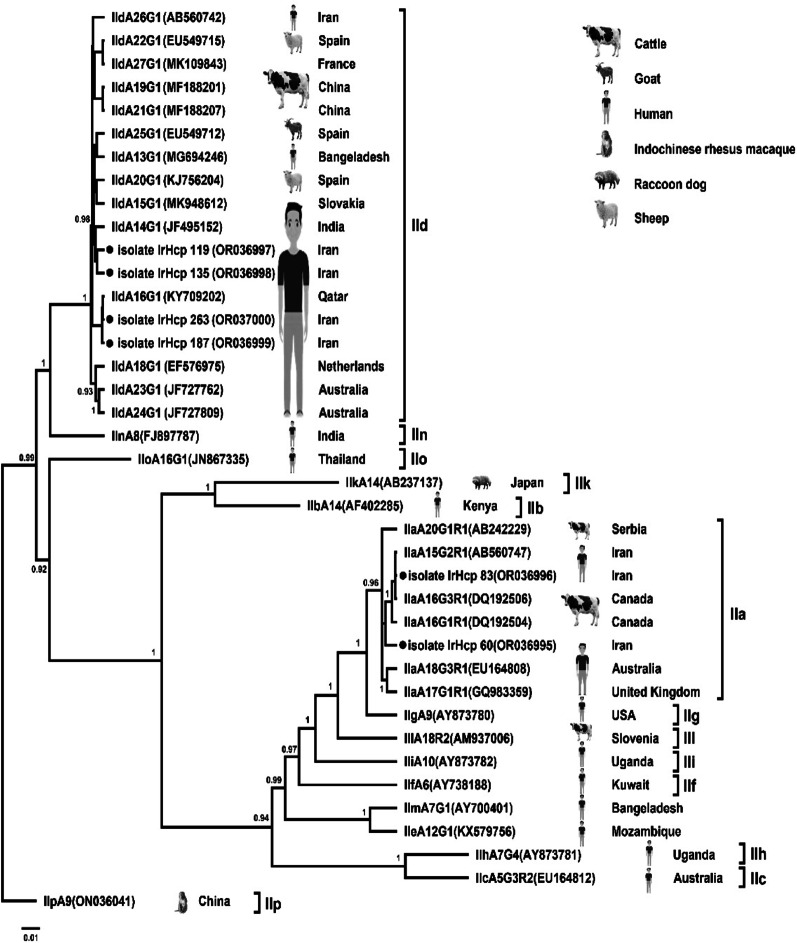
Bayesian phylogenetic tree inferred from the gp60 gene sequences of ***Cryptosporidium parvum*** using BEAST v2.7.7 under the TN93 + G substitution model. Posterior probabilities are displayed on major nodes. The tree demonstrates the clustering of isolates into subtype families IIa and IId, with Iranian isolates (●) mainly belonging to IIdA20G1, IIdA19G1, IIdA20G3R1, and IIaA15G2R1 subtypes. Reference sequences from different geographic origins and hosts are included for comparison

The phylogenetic tree of *C. parvum* based on the gp60 gene revealed distinct clustering into known subtype families, including IIa, IId, IIe, and IIf (Fig. 2). Most Iranian isolates were grouped within the IId family, specifically subtypes IIdA20G1, IIdA19G1, and IIdA20G3R1, which clustered closely with reference sequences from humans and animals in countries such as Iran, China, and India. Posterior probabilities across these clusters ranged from 0.82 to 1.00, confirming robust phylogenetic support. These findings confirm the predominance of zoonotic IId subtypes in this population and suggest regional subtype patterns with potential animal-to-human transmission.

**Fig. 3:**
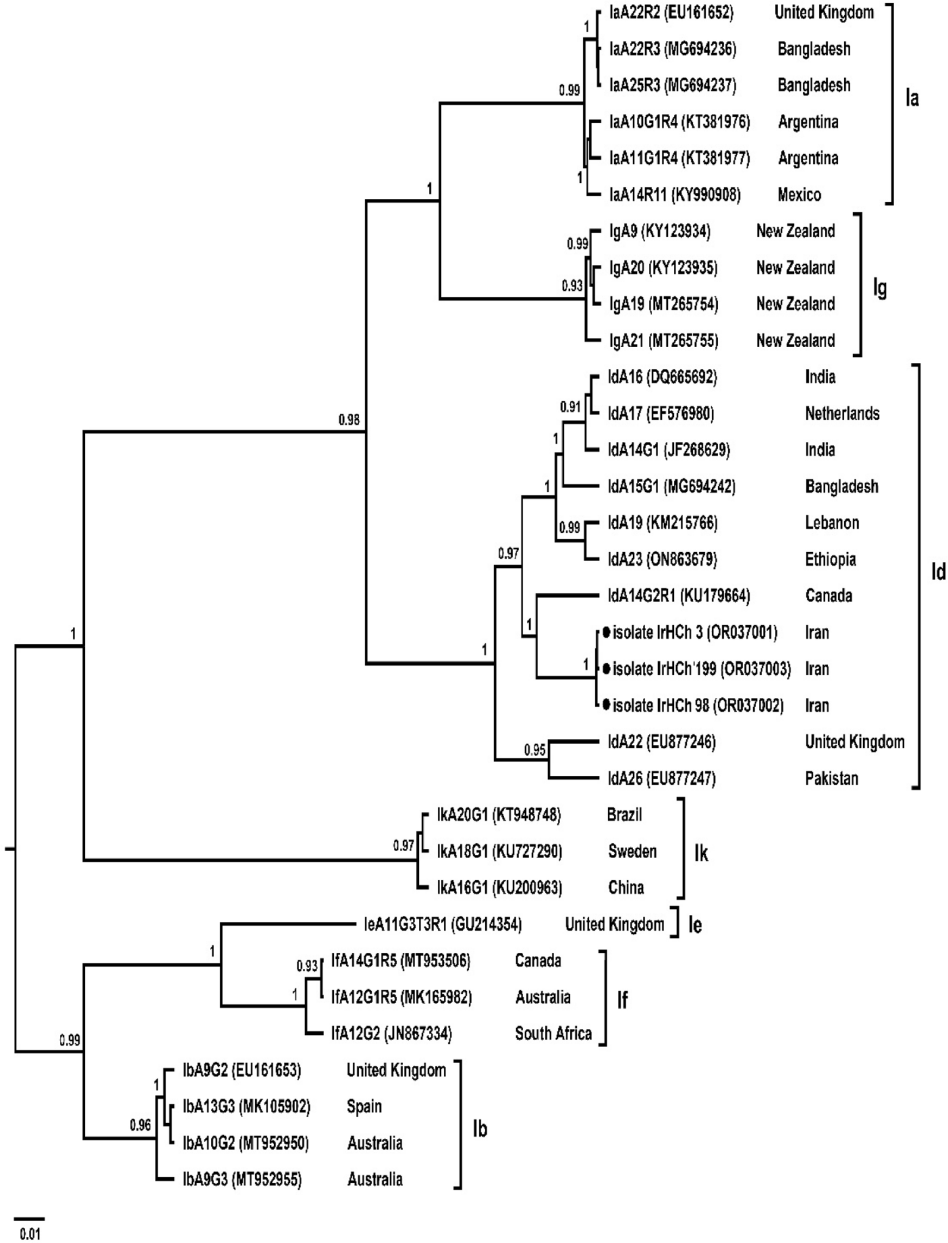
Bayesian phylogenetic tree based on gp60 gene sequences of *Cryptosporidium hominis* constructed using BEAST v2.7.7 under the TN93 + G model. Node values indicate posterior probabilities. All Iranian isolates (●) clustered within the IdA15G1 subtype family, forming a well-supported clade with global reference sequences, suggesting an anthroponotic transmission pattern

For *C. hominis*, all five isolates clustered within the IdA15G1 subtype family in the gp60-based Bayesian tree (Fig. 3). The isolates showed close genetic similarity to sequences reported from Europe and Asia, supporting the global distribution of this subtype and its primarily anthroponotic transmission route. Strong posterior support (≥ 0.9) within this clade indicated low genetic divergence among isolates.

**Fig. 4:**
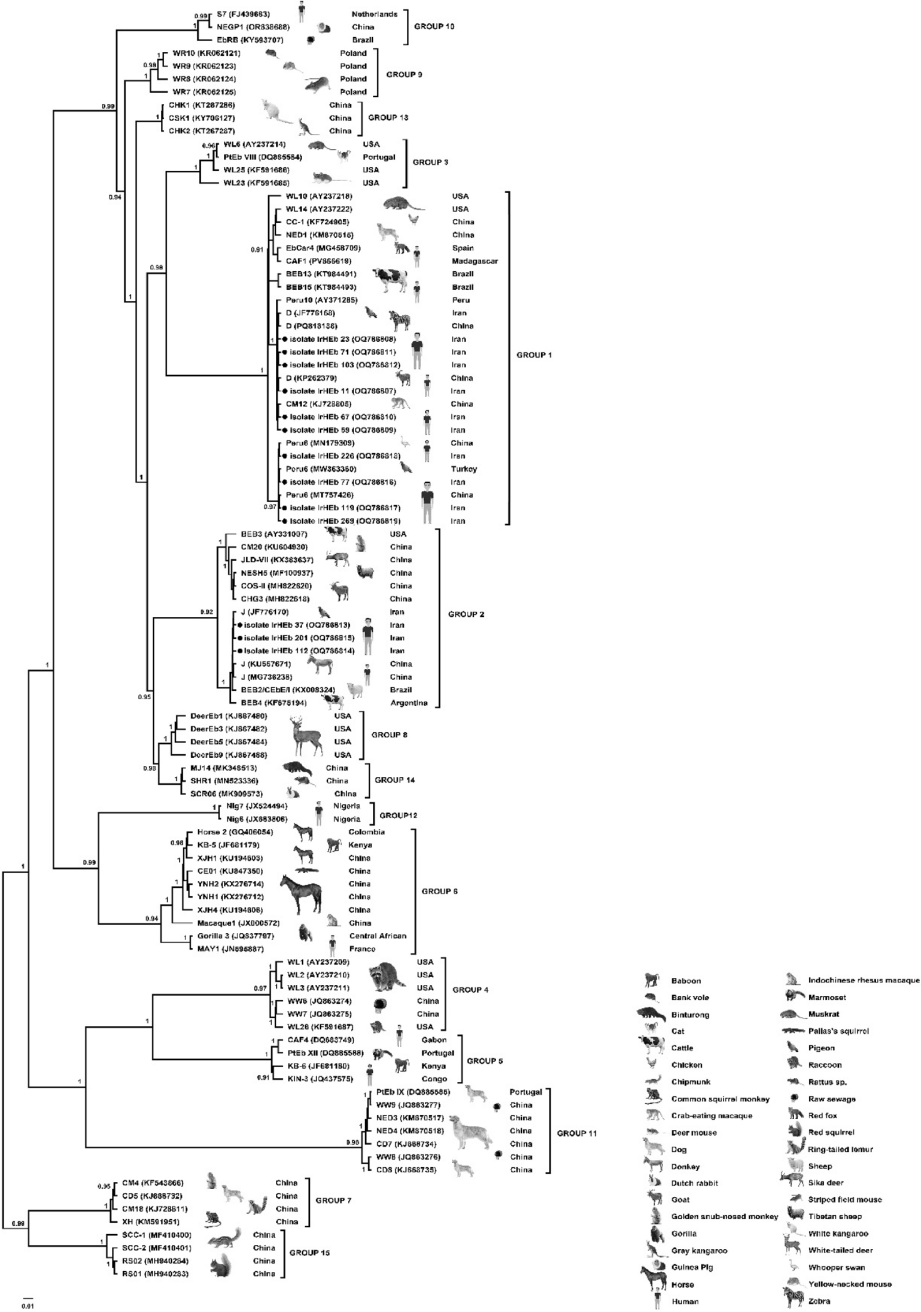
Bayesian phylogenetic tree inferred from Enterocytozoon bieneusi ITS sequences using BEAST v2.7.7 under the TN93 + G model. Posterior probability values are indicated at key nodes. The isolates formed three genotype clusters—D, Peru6, and J (●)—with genotype J representing the first report from HIV-positive patients in Iran. Reference sequences from human and animal hosts are included for phylogenetic comparison. Scale bar represents substitutions per site

Finally, the Bayesian phylogenetic analysis of *Enterocytozoon bieneusi* based on the ITS region revealed three genotype clusters: D, Peru6, and J (Fig. 4). Most isolates belonged to Group 1, which is commonly associated with zoonotic transmission. Genotype J, identified in three patients, formed a distinct, well-supported clade (posterior probability = 0.95) and was reported for the first time in humans in Iran, suggesting a possible local emergence or recent zoonotic spillover. These phylogenetic results corroborate the molecular and epidemiological data, highlighting the genetic diversity of the pathogens and the significance of zoonotic and environmental routes of transmission in this population.

## Discussion

This study highlights the clinical and epidemiological significance of microsporidiosis and cryptosporidiosis in HIV-infected individuals in Alborz, Iran, revealing valuable insights into local transmission dynamics, genotype distribution, and potential risk factors. Despite advancements in antiretroviral therapy (ART), enteric protozoan infections continue to pose a substantial health risk to immunocompromised individuals, particularly in resource-limited settings where diagnostic challenges persist [33].

Our findings confirmed the presence of *E. bieneusi* and *E. intestinalis* in HIV-positive patients, with a higher prevalence of *E. bieneusi*. The predominance of genotype D (*n* = 10) is consistent with previous reports identifying this genotype as the most common in both immunocompetent and immunocompromised individuals globally [34, 35]. The detection of zoonotic genotypes Peru6 (*n* = 5) and J (*n* = 3) further indicates the significance of animal reservoirs in the local transmission cycle [36]. The identification of genotype J in this cohort represents the first report of this genotype in humans in Iran. Although genotype J has previously been detected in cattle within the country [37–39], its presence in human patients provides the first molecular evidence of a potential zoonotic spillover [8]. The Bayesian phylogenetic analysis (BEAST, TN93 + G) showed genotype J forming a distinct, well-supported clade (posterior probability = 0.95), reinforcing its novelty and epidemiological importance in the Iranian context and suggesting a possible recent cross-species transmission event or previously unrecognized local circulation.

The presence of *C. parvum* subtypes IIdA20G1 (*n* = 8), IIdA19G1 (*n* = 4), and IIaA15G2R1 (*n* = 3) reflects a subtype distribution that differs from patterns observed in Western populations, where *C. hominis* is more prevalent [40]. The dominance of IId subtypes aligns with other Middle Eastern and Asian studies that have linked these subtypes to infections from small ruminants such as sheep and goats [41, 42]. The strong association between these subtypes and animal contact or environmental exposure further underscores the zoonotic nature of cryptosporidiosis in this region. Bayesian phylogenetic analysis in BEAST confirmed the predominance of zoonotic IId subtypes, which clustered closely with sequences from both animals and humans in China, India, and Australia, with strong posterior support (0.82–1.00), supporting the hypothesis of cross-species transmission. Meanwhile, *C. hominis* isolates all belonged to the IdA15G1 subtype and formed a tightly clustered clade with reference strains from Europe and Asia (posterior >0.9), suggesting anthroponotic transmission routes.

While zoonotic transmission is strongly supported by the predominance of *C. parvum* IId subtypes and the detection of zoonotic *E. bieneusi* genotypes such as Peru6 and J, other transmission routes should also be considered. The identification of *C. hominis* subtype IdA15G1 in several cases—together with its well-established anthroponotic nature—suggests that human-to-human transmission may occur within households, healthcare facilities, or communities with inadequate sanitation. Moreover, the observed association between well-water use and *C. hominis* infection in this cohort indicates a waterborne pathway, as contaminated groundwater can serve as a vehicle for oocyst dissemination in areas lacking effective water treatment. Consequently, infection-control programs should integrate WASH-based interventions (water, sanitation, and hygiene) alongside zoonotic-control strategies to limit both environmental and interpersonal spread of these opportunistic pathogens.

The identification of *E. bieneusi* genotype J in this cohort represents the first report of this genotype in humans in Iran. Although genotype J has previously been detected in cattle within the country [20–22], its presence in human patients provides the first molecular evidence of a potential zoonotic spillover. The phylogenetic placement of genotype J in a distinct and well-supported clade further reinforces its novelty and epidemiological importance in the Iranian context. From a public health perspective, this emergence underscores the need for targeted One Health investigations to (i) identify animal reservoirs, (ii) assess environmental sources such as drinking water and agricultural runoff, and (iii) conduct clinical surveillance to determine possible genotype-specific outcomes. We recommend expanded molecular monitoring across human, animal, and environmental samples, coupled with comparative genomic analyses to explore virulence markers and public-awareness programs to reduce zoonotic exposure.

From a clinical standpoint, the study observed that infections were not limited to severely immunocompromised individuals. Microsporidial infections occurred even among patients with relatively stable CD4 + T-cell counts and those receiving ART, suggesting persistent exposure and possible gaps in immune protection or reinfection. Importantly, specific genotypes such as *E. bieneusi* genotype D were associated with more severe gastrointestinal symptoms, particularly in HAART-naïve individuals. This observation is supported by recent studies highlighting genotype-dependent virulence and cytokine modulation [43–45].

The overall prevalence of *Cryptosporidium* spp. (7.64% by PCR) and *E. bieneusi* (6.5%) observed in our study falls within the range reported across various regions of Iran. For *Cryptosporidium*, the prevalence among Iranian individuals ranges widely from 0.36% to 12%, with the lowest rates reported in Kurdistan and Hamedan, and the highest in Bandar Abbas and Yazd [46–49]. Similarly, *E. bieneusi* prevalence has been reported from as low as 1.3% to as high as 20%, with lower rates in Tehran (1.3%) and Rasht (2.3%), while higher prevalence has been observed in Tabriz (10.6%), Kerman (13.88%), and Mazandaran (20%) [6, 34, 50–52]. These variations in prevalence may reflect differences in regional environmental conditions, water sources, sanitation, host immunity, and diagnostic methods [53, 54]. Notably, the higher detection rates in our study may be attributed to the use of sensitive molecular techniques, such as nested PCR, which provided more accurate results compared to conventional microscopy, which detected a lower prevalence of *Cryptosporidium* (2.2%).

Our Bayesian phylogenetic analysis based on ITS sequencing revealed that genotype D strains clustered closely with isolates from both humans and animals, including cattle, cat, beaver, chinchilla, primate and dog, from geographically diverse regions such as China, Bangladesh, Thailand, Argentina, Poland, Slovak Republic, Brazil, and Turkey [44, 55–61]. This global clustering highlights the broad host range and high zoonotic potential of genotype D, supporting the One Health perspective [62, 63]. The clear Bayesian phylogenetic separation of *Cryptosporidium* species based on 18 S rRNA sequencing provided additional validation of molecular identification and demonstrated close evolutionary relationships among human-infecting species. The *C. parvum* subtypes identified in this study have been widely reported in livestock in Iran and neighbouring countries, reinforcing the risk of zoonotic transmission in agricultural communities [20, 47, 64–69].

The findings of this study have several public health implications [70]. First, the detection of zoonotic genotypes in patients using well water indicates vulnerabilities in local water treatment infrastructure [71, 72]. Notably, *C. hominis* subtype IdA15G1, known for its resistance to chlorine-based disinfection, was detected in association with well water consumption, echoing previous global reports [73, 74]. These insights call for enhanced surveillance and improved water safety measures, particularly in high-risk areas. Second, the continued presence of these infections in patients on ART suggests that immune reconstitution alone may not be sufficient to prevent infection or that reinfection is occurring. Routine screening of stool samples in HIV patients, especially those with CD4 + counts below 100 cells/µl—could facilitate early diagnosis and intervention. Our data also suggest the presence of asymptomatic carriers who may contribute to ongoing transmission within communities [75, 76].Finally, this study supports the integration of a One Health framework in the control and prevention of protozoan infections, particularly those with zoonotic transmission potential [77]. Coordinated efforts involving veterinary, environmental, and human health sectors are essential to address shared risk factors and implement effective interventions, such as livestock vaccination, environmental sanitation, and public health education [78, 79].

## Conclusion

This study provides molecular and epidemiological evidence for the occurrence of *Cryptosporidium* and *Microsporidia* infections among HIV-positive individuals in Alborz, Iran. The identification of zoonotic genotypes and subtypes—particularly *C. parvum* IIdA20G1 and *E. bieneusi* genotype J—highlights the ongoing risk of cross-species transmission in this vulnerable population. Bayesian phylogenetic analyses (BEAST, TN93 + G model) confirmed the genetic relatedness of Iranian isolates to strains from both human and animal sources worldwide, underscoring the importance of a One Health approach in surveillance and control strategies. The associations between infections and environmental exposure, immunosuppression, and lack of antiretroviral therapy further emphasize the multifactorial nature of infection risk. These findings call for integrated public health interventions including improved water safety, targeted screening in immunocompromised patients, and strengthened collaboration across human, veterinary, and environmental health sectors to mitigate the burden of these opportunistic infections in endemic settings.

## Data Availability

The data that support the findings of this study is available from the corresponding author on reasonable request.

## Abbreviations

HIV: Human immunodeficiency virus
UNAIDS: United nations program on HIV/AIDS
CTAB: Modified cetyltrimethylammonium bromide
MEGA: Molecular evolutionary genetics analysis
BLAST: Basic local alignment search tool
NCBI: National centre for biotechnology information
ART: Antiretroviral therapy
HAART: Highly active antiretroviral therapy
